# Supplementary material for: A novel ciprofloxacin-resistant subclade of H58 Salmonella Typhi is associated with fluoroquinolone treatment failure
Source: eLife. 2016 Mar 14;5:e14003. doi: 10.7554/eLife.14003 (PMC4805543; doi:10.7554/eLife.14003)
Supplement: Supplementary file 1. — DOI: http://dx.doi.org/10.7554/eLife.14003.010 [file elife-14003-supp1.docx]

**Supplementary file 1** Table of *Salmonella* Typhi isolates and their corresponding sequencing metadata used in this study

| **Name** | **Tree ID** | **Accession number** | **Trial** | **Year** | **Country** | **gyrA**  **S83F** | **gyrA**  **D87N** | **gyrA**  **D87V** | **gyrB**  **S464Y** | **gyrB**  **A574V** | **parC**  **S80I** | **parE**  **A353V** | **parE**  **A364V** | **parE**  **E460K** | **H58** | **MIC**  **Cipro** | **MIC group*** |
| --- | --- | --- | --- | --- | --- | --- | --- | --- | --- | --- | --- | --- | --- | --- | --- | --- | --- |
| 02TY_001 | STY1_AL513382 | ERR1079230 | 1 | 2011 | Nepal | 0 | 0 | 0 | 0 | 0 | 0 | 0 | 0 | 0 | 1 | 0·016 | 1 |
| 02TY_006 | STY6_AL513382 | ERR1079231 | 1 | 2011 | Nepal | 1 | 0 | 0 | 0 | 0 | 0 | 0 | 0 | 0 | 1 | 0·38 | 2 |
| 02TY_007 | STY7_AL513382 | ERR1079232 | 1 | 2011 | Nepal | 1 | 0 | 0 | 0 | 0 | 0 | 0 | 0 | 0 | 1 | 0·25 | 2 |
| 02TY_009 | 13STY9_AL513382 | ERR1079233 | 1 | 2011 | Nepal | 1 | 0 | 0 | 0 | 0 | 0 | 0 | 0 | 0 | 1 | 0·38 | 2 |
| 02TY_015 | STY15_MERGE_AL513382 | ERR1079234 | 1 | 2012 | Nepal | 1 | 0 | 0 | 0 | 0 | 0 | 0 | 0 | 0 | 1 | 0·38 | 2 |
| 02TY_016 | STY16_AL513382 | ERR1079235 | 1 | 2012 | Nepal | 1 | 0 | 0 | 0 | 0 | 0 | 0 | 0 | 0 | 1 | 0·38 | 2 |
| 02TY_018 | STY18_AL513382 | ERR1079236 | 1 | 2012 | Nepal | 1 | 0 | 0 | 0 | 0 | 0 | 0 | 0 | 0 | 1 | 0·38 | 2 |
| 02TY_027 | STY27_MERGE_AL513382 | ERR1079237 | 1 | 2012 | Nepal | 1 | 0 | 0 | 0 | 0 | 0 | 0 | 0 | 0 | 1 | 0·38 | 2 |
| 02TY_028 | STY28_MERGE_AL513382 | ERR1079238 | 1 | 2012 | Nepal | 1 | 0 | 0 | 0 | 0 | 0 | 0 | 0 | 0 | 1 | 0·38 | 2 |
| 02TY_030 | STY30_AL513382 | ERR1079239 | 1 | 2012 | Nepal | 1 | 0 | 0 | 0 | 0 | 0 | 0 | 0 | 0 | 1 | 0·38 | 2 |
| 02TY_031 | STY31_AL513382 | ERR1079240 | 1 | 2012 | Nepal | 1 | 0 | 0 | 0 | 0 | 0 | 0 | 0 | 0 | 1 | 0·38 | 2 |
| 02TY_032 | STY32_AL513382 | ERR1079241 | 1 | 2012 | Nepal | 1 | 0 | 0 | 0 | 0 | 0 | 0 | 0 | 0 | 1 | 0·25 | 2 |
| 02TY_034 | STY34_AL513382 | ERR1079242 | 1 | 2012 | Nepal | 1 | 0 | 0 | 0 | 0 | 0 | 0 | 0 | 0 | 1 | 0·5 | 2 |
| 02TY_037 | STY37_AL513382 | ERR1079243 | 1 | 2012 | Nepal | 1 | 0 | 0 | 0 | 0 | 0 | 0 | 0 | 0 | 1 | 0·25 | 2 |
| 02TY_038 | STY38_AL513382 | ERR1079244 | 1 | 2012 | Nepal | 1 | 0 | 0 | 0 | 0 | 0 | 0 | 0 | 0 | 1 | 0·38 | 2 |
| 02TY_045 | STY45_MERGE_AL513382 | ERR1079245 | 1 | 2012 | Nepal | 1 | 0 | 0 | 0 | 0 | 0 | 0 | 0 | 0 | 1 | 0·25 | 2 |
| 02TY_047 | STY47_AL513382 | ERR1079246 | 1 | 2012 | Nepal | 1 | 0 | 0 | 0 | 0 | 0 | 0 | 0 | 0 | 1 | 0·38 | 2 |
| 02TY_048 | STY48_AL513382 | ERR1079247 | 1 | 2012 | Nepal | 1 | 0 | 0 | 0 | 0 | 0 | 0 | 0 | 0 | 1 | 0·125 | 2 |
| 02TY_051 | STY51_AL513382 | ERR1079248 | 1 | 2012 | Nepal | 1 | 0 | 0 | 0 | 0 | 0 | 0 | 0 | 0 | 1 | 0·25 | 2 |
| 02TY_055 | STY55_AL513382 | ERR1079249 | 1 | 2012 | Nepal | 1 | 0 | 0 | 0 | 0 | 0 | 0 | 0 | 0 | 1 | 0·25 | 2 |
| 02TY_057 | STY57_AL513382 | ERR1079250 | 1 | 2012 | Nepal | 1 | 0 | 0 | 0 | 0 | 0 | 0 | 0 | 0 | 1 | 0·25 | 2 |
| 02TY_058 | 4STY58D1_AL513382 | ERR1079251 | 1 | 2012 | Nepal | 1 | 0 | 0 | 0 | 0 | 0 | 0 | 0 | 0 | 1 | 0·25 | 2 |
| 02TY_059 | STY59_AL513382 | ERR1079253 | 1 | 2012 | Nepal | 1 | 0 | 0 | 0 | 0 | 0 | 0 | 0 | 0 | 1 | 0·25 | 2 |
| 02TY_060 | STY60_AL513382 | ERR1079254 | 1 | 2012 | Nepal | 1 | 0 | 0 | 0 | 0 | 0 | 0 | 0 | 0 | 1 | 0·19 | 2 |
| 02TY_061 | STY61_AL513382 | ERR1079255 | 1 | 2012 | Nepal | 0 | 0 | 0 | 0 | 0 | 0 | 0 | 1 | 0 | 0 | 0·012 | 1 |
| 02TY_078 | 14STY78_AL513382 | ERR1079256 | 1 | 2012 | Nepal | 0 | 0 | 0 | 0 | 0 | 0 | 0 | 0 | 0 | 0 | 0·016 | 1 |
| 02TY_080 | STY80_AL513382 | ERR1079257 | 1 | 2012 | Nepal | 1 | 0 | 0 | 0 | 0 | 0 | 0 | 0 | 0 | 1 | 0·19 | 2 |
| 02TY_083 | STY83_AL513382 | ERR1079258 | 1 | 2012 | Nepal | 1 | 0 | 0 | 0 | 0 | 0 | 0 | 0 | 0 | 1 | 0·5 | 2 |
| 02TY_119 | 15STY119D1_AL513382 | ERR1079279 | 1 | 2013 | Nepal | 1 | 0 | 0 | 0 | 0 | 0 | 0 | 0 | 0 | 1 | 0·25 | 2 |
| 02TY_125 | STY125_MERGE_AL513382 | ERR1079260 | 1 | 2013 | Nepal | 1 | 0 | 0 | 0 | 0 | 0 | 0 | 0 | 0 | 1 | 0·38 | 2 |
| 02TY_126 | 6STY126_AL513382 | ERR1079261 | 1 | 2013 | Nepal | 1 | 0 | 0 | 0 | 0 | 0 | 0 | 0 | 0 | 1 | 0·25 | 2 |
| 02TY_127 | 7STY127_AL513382 | ERR1079262 | 1 | 2013 | Nepal | 0 | 1 | 0 | 0 | 0 | 0 | 0 | 0 | 0 | 0 | 0·125 | 2 |
| 02TY_132 | 15STY132_AL513382 | ERR1079263 | 1 | 2013 | Nepal | 1 | 1 | 0 | 0 | 0 | 1 | 0 | 0 | 0 | 1 | 32 | 3 |
| 02TY_135 | 16STY135_AL513382 | ERR1079264 | 1 | 2013 | Nepal | 1 | 0 | 0 | 0 | 0 | 0 | 0 | 0 | 0 | 1 | 0·25 | 2 |
| 02TY_136 | STY136_MERGE_AL513382 | ERR1079265 | 1 | 2013 | Nepal | 0 | 0 | 0 | 0 | 0 | 0 | 0 | 0 | 0 | 1 | 0·023 | 1 |
| 02TY_140 | 16STY140_AL513382 | ERR1079266 | 1 | 2013 | Nepal | 0 | 0 | 0 | 0 | 0 | 0 | 0 | 0 | 0 | 1 | 0·023 | 1 |
| 02TY_143 | 9STY143D26_AL513382 | ERR1079267 | 1 | 2013 | Nepal | 1 | 0 | 0 | 0 | 0 | 0 | 0 | 0 | 0 | 1 | 0·25 | 2 |
| 02TY_144 | 10STY144_AL513382 | ERR1079268 | 1 | 2013 | Nepal | 1 | 0 | 0 | 0 | 0 | 0 | 0 | 0 | 0 | 1 | 0·19 | 2 |
| 02TY_146 | 11STY146_AL513382 | ERR1079269 | 1 | 2013 | Nepal | 1 | 0 | 0 | 0 | 0 | 0 | 0 | 0 | 0 | 1 | 0·25 | 2 |
| 02TY_147 | 17STY147_AL513382 | ERR1079270 | 1 | 2013 | Nepal | 1 | 0 | 0 | 0 | 1 | 0 | 0 | 1 | 0 | 0 | 1 | 3 |
| 02TY_148 | 17STY148_AL513382 | ERR1079271 | 1 | 2013 | Nepal | 1 | 0 | 0 | 0 | 1 | 0 | 0 | 1 | 0 | 0 | 0·38 | 2 |
| 02TY_149 | 12STY149_AL513382 | ERR1079272 | 1 | 2013 | Nepal | 1 | 0 | 0 | 0 | 0 | 0 | 0 | 0 | 0 | 1 | 0·38 | 2 |
| 02TY_150 | 13STY150_AL513382 | ERR1079273 | 1 | 2013 | Nepal | 1 | 0 | 0 | 0 | 0 | 0 | 0 | 0 | 0 | 1 | 0·25 | 2 |
| 02TY_156 | 14STY156_AL513382 | ERR1079274 | 1 | 2013 | Nepal | 1 | 0 | 0 | 0 | 0 | 0 | 0 | 0 | 0 | 1 | 0·38 | 2 |
| 02TY_157 | 18STY157_AL513382 | ERR1079275 | 1 | 2013 | Nepal | 0 | 0 | 0 | 0 | 0 | 0 | 1 | 1 | 0 | 0 | 0·008 | 1 |
| 02TY_158 | 15STY158_AL513382 | ERR1079276 | 1 | 2013 | Nepal | 1 | 0 | 0 | 0 | 0 | 0 | 0 | 0 | 0 | 1 | 0·38 | 2 |
| 02TY_159 | 16STY159_AL513382 | ERR1079277 | 1 | 2013 | Nepal | 1 | 0 | 0 | 0 | 0 | 0 | 0 | 0 | 0 | 1 | 1 | 3 |
| 02TY_160 | 17STY160_AL513382 | ERR1079278 | 1 | 2013 | Nepal | 1 | 1 | 0 | 0 | 0 | 1 | 0 | 0 | 0 | 1 | 32 | 3 |
| 02TY_163 | 18STY163_AL513382 | ERR1079279 | 1 | 2013 | Nepal | 1 | 1 | 0 | 0 | 0 | 1 | 0 | 0 | 0 | 1 | 32 | 3 |
| 02TY_164 | 19STY164_AL513382 | ERR1079280 | 1 | 2013 | Nepal | 0 | 0 | 0 | 0 | 0 | 0 | 0 | 0 | 0 | 0 | 0·016 | 1 |
| 02TY_169 | STY169_MERGE_AL513382 | ERR1079281 | 1 | 2013 | Nepal | 1 | 1 | 0 | 0 | 0 | 1 | 0 | 0 | 0 | 1 | 24 | 3 |
| 02TY_173 | STY173_MERGE_AL513382 | ERR1079282 | 1 | 2013 | Nepal | 1 | 0 | 0 | 0 | 0 | 0 | 0 | 0 | 0 | 1 | 0·38 | 2 |
| 02TY_174 | 19STY174_AL513382 | ERR1079283 | 1 | 2013 | Nepal | 1 | 0 | 1 | 0 | 0 | 1 | 0 | 1 | 0 | 0 | 32 | 3 |
| 02TY_176 | 22STY176D1_AL513382 | ERR1079284 | 1 | 2013 | Nepal | 1 | 1 | 0 | 0 | 0 | 1 | 0 | 0 | 0 | 1 | 32 | 3 |
| 02TY_178 | 24STY178_AL513382 | ERR1079286 | 1 | 2013 | Nepal | 1 | 0 | 1 | 0 | 0 | 1 | 0 | 1 | 0 | 0 | 32 | 3 |
| 02TY_183 | 13254_5#37 | ERR586912 | 1 | 2013 | Nepal | 1 | 1 | 0 | 0 | 0 | 1 | 0 | 0 | 0 | 1 | 32 | 3 |
| 02TY_185 | 25STY185_AL513382 | ERR1079287 | 1 | 2013 | Nepal | 0 | 0 | 0 | 1 | 0 | 0 | 0 | 0 | 0 | 0 | 0·15 | 2 |
| 02TY_186 | 26STY186_AL513382 | ERR1079288 | 1 | 2013 | Nepal | 1 | 0 | 0 | 0 | 0 | 0 | 0 | 0 | 0 | 1 | 0·25 | 2 |
| 02TY_188 | STY188_MERGE_AL513382 | ERR1079289 | 1 | 2013 | Nepal | 1 | 1 | 0 | 0 | 0 | 1 | 0 | 0 | 0 | 1 | 32 | 3 |
| 02TY_190 | 13254_5#39 | ERR586914 | 1 | 2013 | Nepal | 1 | 1 | 0 | 0 | 0 | 1 | 0 | 0 | 0 | 1 | 24 | 3 |
| 02TY_195 | STY195_MERGE_AL513382 | ERR1079290 | 1 | 2013 | Nepal | 1 | 0 | 0 | 0 | 0 | 0 | 0 | 0 | 0 | 1 | 0·19 | 2 |
| 02TY_196 | 29STY196_AL513382 | ERR1079291 | 1 | 2013 | Nepal | 0 | 0 | 0 | 0 | 0 | 0 | 0 | 0 | 0 | 1 | 0·016 | 1 |
| 02TY_200 | 30STY200_AL513382 | ERR1079292 | 1 | 2013 | Nepal | 0 | 0 | 0 | 0 | 0 | 0 | 0 | 0 | 0 | 0 | 0·016 | 1 |
| 02TY_202 | 20STY202_AL513382 | ERR1079293 | 1 | 2013 | Nepal | 1 | 0 | 0 | 0 | 0 | 0 | 0 | 1 | 0 | 0 | 0·25 | 2 |
| 02TY_210 | 18STY210_AL513382 | ERR1079294 | 1 | 2013 | Nepal | 1 | 1 | 0 | 0 | 0 | 1 | 0 | 0 | 0 | 1 | 32 | 3 |
| 02TY_213 | 24STY213_AL513382 | ERR1079295 | 1 | 2014 | Nepal | 0 | 0 | 0 | 0 | 0 | 0 | 0 | 1 | 0 | 0 | 0·016 | 1 |
| 02TY_216 | 31STY216_AL513382 | ERR1079296 | 1 | 2014 | Nepal | 1 | 0 | 0 | 0 | 0 | 0 | 0 | 0 | 0 | 1 | 0·25 | 2 |
| 02TY_219 | 21STY219D1_AL513382 | ERR1079297 | 1 | 2014 | Nepal | 1 | 1 | 0 | 0 | 0 | 1 | 0 | 0 | 0 | 1 | 32 | 3 |
| 02TY_222 | 23STY222_AL513382 | ERR1079299 | 1 | 2014 | Nepal | 1 | 0 | 0 | 0 | 0 | 0 | 0 | 0 | 1 | 1 | 0·38 | 2 |
| 02TY_226 | 32STY226_AL513382 | ERR1079300 | 1 | 2014 | Nepal | 1 | 0 | 0 | 0 | 0 | 0 | 0 | 0 | 0 | 1 | 0·25 | 2 |
| 02TY_229 | 20STY229_AL513382 | ERR1079301 | 1 | 2014 | Nepal | 1 | 1 | 0 | 0 | 0 | 1 | 0 | 0 | 0 | 1 | 32 | 3 |
| 02TY_232 | STY232_MERGE_AL513382 | ERR1079302 | 1 | 2014 | Nepal | 1 | 1 | 0 | 0 | 0 | 1 | 0 | 0 | 0 | 1 | 32 | 3 |
| 02TY_235 | 24STY235D1_AL513382 | ERR1079303 | 1 | 2014 | Nepal | 1 | 0 | 0 | 0 | 0 | 0 | 0 | 0 | 0 | 1 | 0·38 | 2 |
| 02TY_236 | STY236_MERGE_AL513382 | ERR1079305 | 1 | 2014 | Nepal | 1 | 0 | 0 | 0 | 0 | 0 | 0 | 0 | 0 | 1 | 0·38 | 2 |
| 02TY_239 | 28STY239_AL513382 | ERR1079306 | 1 | 2014 | Nepal | 1 | 0 | 0 | 0 | 0 | 0 | 0 | 0 | 0 | 1 | 0·25 | 2 |
| 02TY_240 | 29STY240_AL513382 | ERR1079307 | 1 | 2014 | Nepal | 1 | 0 | 0 | 0 | 0 | 0 | 0 | 0 | 0 | 1 | 0·25 | 2 |
| 02TY_241 | 30STY241_AL513382 | ERR1079308 | 1 | 2014 | Nepal | 1 | 0 | 0 | 0 | 0 | 0 | 0 | 0 | 0 | 1 | 0·5 | 2 |
| 02TY_244 | 12STY244_AL513382 | ERR1079309 | 1 | 2014 | Nepal | 1 | 0 | 0 | 0 | 0 | 0 | 0 | 0 | 0 | 1 | 0·25 | 2 |
| 01TY072 | 5886_3#1 | ERR119817 | 0 | 2008 | Nepal | 0 | 0 | 0 | 0 | 0 | 0 | 0 | 0 | 0 | 0 | 0·016 | 1 |
| 01TY075 | 5886_3#2 | ERR119818 | 0 | 2008 | Nepal | 0 | 0 | 0 | 0 | 0 | 0 | 0 | 0 | 0 | 0 | 0·016 | 1 |
| 01TY090 | 5886_3#4 | ERR119820 | 0 | 2009 | Nepal | 0 | 0 | 0 | 0 | 0 | 0 | 0 | 0 | 0 | 1 | 0·016 | 1 |
| 01TY098 | 5886_3#5 | ERR119821 | 0 | 2009 | Nepal | 0 | 0 | 0 | 0 | 0 | 0 | 0 | 0 | 0 | 0 | 0·016 | 1 |
| 01TY101 | 5886_3#6 | ERR119822 | 0 | 2009 | Nepal | 0 | 0 | 0 | 0 | 0 | 0 | 0 | 0 | 0 | 1 | 0·016 | 1 |
| 01TY103 | 5886_3#3 | ERR119819 | 0 | 2009 | Nepal | 0 | 0 | 0 | 0 | 0 | 0 | 0 | 0 | 0 | 1 | 0·016 | 1 |
| 01TY104 | 5886_3#7 | ERR119823 | 0 | 2009 | Nepal | 0 | 0 | 0 | 0 | 0 | 0 | 0 | 0 | 0 | 1 | 0·016 | 1 |
| 01TY112 | 5886_3#8 | ERR119824 | 0 | 2009 | Nepal | 0 | 0 | 0 | 0 | 0 | 0 | 0 | 0 | 0 | 1 | 0·016 | 1 |
| 01TY122 | 5886_3#9 | ERR119825 | 0 | 2009 | Nepal | 0 | 0 | 0 | 0 | 0 | 0 | 0 | 0 | 0 | 1 | 0·016 | 1 |
| CC_04 | 10561_2#1 | ERR357576 | 0 | 2011 | Nepal | 1 | 0 | 0 | 0 | 0 | 0 | 0 | 0 | 0 | 1 | 0·19 | 2 |
| CC_05 | 10561_2#8 | ERR357583 | 0 | 2011 | Nepal | 1 | 0 | 0 | 0 | 0 | 0 | 0 | 0 | 0 | 1 | 0·19 | 2 |
| CC_08 | 10561_2#12 | ERR357587 | 0 | 2011 | Nepal | 1 | 0 | 0 | 0 | 0 | 0 | 0 | 0 | 0 | 1 | 0·19 | 2 |
| CC_11 | 10561_2#19 | ERR357594 | 0 | 2011 | Nepal | 1 | 0 | 0 | 0 | 0 | 0 | 0 | 0 | 0 | 1 | 0·25 | 2 |
| CC_12 | 10561_2#25 | ERR357600 | 0 | 2011 | Nepal | 0 | 0 | 0 | 0 | 0 | 0 | 0 | 1 | 0 | 0 | 0·004 | 1 |
| CC_13 | 10561_2#31 | ERR357606 | 0 | 2011 | Nepal | 1 | 0 | 0 | 0 | 0 | 0 | 0 | 0 | 0 | 1 | 0·19 | 2 |
| CC_16 | 10561_2#37 | ERR357612 | 0 | 2011 | Nepal | 0 | 0 | 0 | 0 | 0 | 0 | 0 | 1 | 0 | 0 | 0·004 | 1 |
| CC_22 | 10561_2#44 | ERR357619 | 0 | 2011 | Nepal | 1 | 0 | 0 | 0 | 0 | 0 | 0 | 0 | 0 | 1 | 0·25 | 2 |
| CC_27 | 10425_1#9 | ERR349339 | 0 | 2011 | Nepal | 1 | 0 | 0 | 0 | 0 | 0 | 0 | 0 | 0 | 1 | 0·38 | 2 |
| CC_31 | 10561_2#53 | ERR357628 | 0 | 2011 | Nepal | 1 | 0 | 0 | 0 | 0 | 0 | 0 | 0 | 0 | 1 | 0·25 | 2 |
| CC_39 | 10561_2#57 | ERR357632 | 0 | 2011 | Nepal | 1 | 0 | 0 | 0 | 0 | 0 | 0 | 0 | 0 | 1 | 0·25 | 2 |
| CC_40 | 10561_2#62 | ERR357637 | 0 | 2011 | Nepal | 1 | 0 | 0 | 0 | 0 | 0 | 0 | 0 | 0 | 1 | 0·38 | 2 |
| CC_41 | 10561_2#2 | ERR357577 | 0 | 2011 | Nepal | 1 | 0 | 0 | 0 | 0 | 0 | 0 | 1 | 0 | 0 | 0·125 | 2 |
| CC_43 | 10561_2#9 | ERR357584 | 0 | 2011 | Nepal | 1 | 0 | 0 | 0 | 0 | 0 | 0 | 0 | 0 | 1 | 0·38 | 2 |
| CC_44 | 10561_2#13 | ERR357588 | 0 | 2011 | Nepal | 1 | 0 | 0 | 0 | 0 | 0 | 0 | 0 | 0 | 1 | 0·25 | 2 |
| CC_45 | 10425_1#5 | ERR349335 | 0 | 2011 | Nepal | 0 | 0 | 0 | 0 | 0 | 0 | 0 | 1 | 0 | 0 | 0·008 | 1 |
| CC_49 | 10561_2#26 | ERR357601 | 0 | 2011 | Nepal | 1 | 0 | 0 | 0 | 0 | 0 | 0 | 0 | 0 | 1 | 0·25 | 2 |
| CC_50 | 10561_2#32 | ERR357607 | 0 | 2011 | Nepal | 0 | 1 | 0 | 0 | 0 | 0 | 0 | 0 | 0 | 0 | 0·125 | 2 |
| CC_51 | 10561_2#38 | ERR357613 | 0 | 2011 | Nepal | 1 | 0 | 0 | 0 | 0 | 0 | 0 | 0 | 0 | 1 | 0·38 | 2 |
| CC_57 | 10561_2#45 | ERR357620 | 0 | 2011 | Nepal | 1 | 0 | 0 | 0 | 0 | 0 | 0 | 0 | 0 | 1 | 0·38 | 2 |
| CC_60 | 10561_2#50 | ERR357625 | 0 | 2011 | Nepal | 1 | 0 | 0 | 0 | 0 | 0 | 0 | 0 | 0 | 1 | 0·19 | 2 |
| CC_61 | 10426_1#6 | ERR349528 | 0 | 2011 | Nepal | 1 | 0 | 0 | 0 | 0 | 0 | 0 | 0 | 0 | 1 | 0·19 | 2 |
| CC_65 | 10561_2#58 | ERR357633 | 0 | 2011 | Nepal | 1 | 0 | 0 | 0 | 0 | 0 | 0 | 0 | 0 | 1 | 0·125 | 2 |
| CC_67 | 10561_2#63 | ERR357638 | 0 | 2011 | Nepal | 1 | 0 | 0 | 0 | 0 | 0 | 0 | 0 | 0 | 1 | 0·19 | 2 |
| CC_69 | 10425_1#2 | ERR349332 | 0 | 2011 | Nepal | 1 | 0 | 0 | 0 | 0 | 0 | 0 | 0 | 0 | 1 | 0·125 | 2 |
| CC_74 | 10425_1#3 | ERR349333 | 0 | 2011 | Nepal | 1 | 0 | 0 | 0 | 0 | 0 | 0 | 0 | 0 | 1 | 0·25 | 2 |
| CC_75 | 10561_2#14 | ERR357589 | 0 | 2011 | Nepal | 1 | 0 | 0 | 0 | 0 | 0 | 0 | 0 | 0 | 1 | 0·25 | 2 |
| CC_80 | 10425_1#6 | ERR349336 | 0 | 2011 | Nepal | 1 | 0 | 0 | 0 | 0 | 0 | 0 | 0 | 0 | 1 | 0·19 | 2 |
| CC_81 | 10561_2#27 | ERR357602 | 0 | 2011 | Nepal | 1 | 0 | 0 | 0 | 0 | 0 | 0 | 0 | 0 | 1 | 0·19 | 2 |
| CC_83 | 10561_2#33 | ERR357608 | 0 | 2011 | Nepal | 1 | 0 | 0 | 0 | 0 | 0 | 0 | 0 | 0 | 1 | 0·25 | 2 |
| CC_87 | 10561_2#39 | ERR357614 | 0 | 2011 | Nepal | 1 | 0 | 0 | 0 | 0 | 0 | 0 | 0 | 0 | 1 | 0·25 | 2 |
| CC_88 | 10561_2#46 | ERR357621 | 0 | 2011 | Nepal | 1 | 0 | 0 | 0 | 0 | 0 | 0 | 0 | 0 | 1 | 0·19 | 2 |
| CC_89 | 10561_2#51 | ERR357626 | 0 | 2011 | Nepal | 1 | 0 | 0 | 0 | 0 | 0 | 0 | 0 | 0 | 1 | 0·19 | 2 |
| CC_90 | 10561_2#54 | ERR357629 | 0 | 2011 | Nepal | 1 | 0 | 0 | 0 | 0 | 0 | 0 | 0 | 0 | 1 | 0·19 | 2 |
| CC_92 | 10561_2#59 | ERR357634 | 0 | 2011 | Nepal | 1 | 0 | 0 | 0 | 0 | 0 | 0 | 0 | 0 | 1 | 0·19 | 2 |
| CC_93 | 10561_2#64 | ERR357639 | 0 | 2011 | Nepal | 1 | 0 | 0 | 0 | 0 | 0 | 0 | 0 | 0 | 1 | 0·19 | 2 |
| CC_94 | 10561_2#3 | ERR357578 | 0 | 2011 | Nepal | 1 | 0 | 0 | 0 | 0 | 0 | 0 | 0 | 0 | 1 | 0·19 | 2 |
| CC_95 | 10561_2#10 | ERR357585 | 0 | 2011 | Nepal | 1 | 0 | 0 | 0 | 0 | 0 | 0 | 0 | 0 | 1 | 0·19 | 2 |
| CC_97 | 10561_2#15 | ERR357590 | 0 | 2011 | Nepal | 1 | 0 | 0 | 0 | 0 | 0 | 0 | 0 | 0 | 1 | 0·19 | 2 |
| CC_99 | 10561_2#20 | ERR357595 | 0 | 2011 | Nepal | 1 | 0 | 0 | 0 | 0 | 0 | 0 | 1 | 0 | 0 | 0·094 | 2 |
| Ind082356_2008 | 10349_1#34 | ERR343282 | 0 | 2008 | India | 1 | 1 | 0 | 0 | 0 | 1 | 0 | 0 | 0 | 1 | 32 | 3 |
| Ind101104_2010 | 10349_1#52 | ERR343300 | 0 | 2010 | India | 1 | 1 | 0 | 0 | 0 | 1 | 0 | 0 | 0 | 1 | 32 | 3 |
| IndBCR175_2011 | 8616_4#48 | ERR420424 | 0 | 2011 | India | 1 | 1 | 0 | 0 | 0 | 1 | 0 | 0 | 0 | 1 | 32 | 3 |
| IndBCR191_2011 | 8616_4#50 | ERR420426 | 0 | 2011 | India | 1 | 1 | 0 | 0 | 0 | 1 | 0 | 0 | 0 | 1 | 32 | 3 |
| IndBCR211_2011 | 8616_4#51 | ERR420427 | 0 | 2011 | India | 1 | 1 | 0 | 0 | 0 | 1 | 0 | 0 | 0 | 1 | 32 | 3 |
| IndBCR62_2009 | 8616_4#41 | ERR420417 | 0 | 2009 | India | 1 | 1 | 0 | 0 | 0 | 1 | 0 | 0 | 0 | 1 | 32 | 3 |
| IndMDUST127_2011 | 10492_1#6 | ERR352259 | 0 | 2011 | India | 1 | 1 | 0 | 0 | 0 | 1 | 0 | 0 | 0 | 1 | 32 | 3 |
| IndMDUST128_2011 | 10493_1#9 | ERR352434 | 0 | 2011 | India | 1 | 1 | 0 | 0 | 0 | 1 | 0 | 0 | 0 | 1 | 32 | 3 |
| IndMDUST147_2012 | 10492_1#13 | ERR352266 | 0 | 2012 | India | 1 | 1 | 0 | 0 | 0 | 1 | 0 | 0 | 0 | 1 | 32 | 3 |
| IndMDUST167_2011 | 10562_2#13 | ERR357768 | 0 | 2011 | India | 1 | 1 | 0 | 0 | 0 | 1 | 0 | 0 | 0 | 1 | 24 | 3 |
| IndMDUST197_2010 | 10492_1#25 | ERR352278 | 0 | 2010 | India | 1 | 1 | 0 | 0 | 0 | 1 | 0 | 0 | 0 | 1 | 32 | 3 |
| IndMDUST216_2011 | 10492_1#31 | ERR352284 | 0 | 2011 | India | 1 | 1 | 0 | 0 | 0 | 1 | 0 | 0 | 0 | 1 | 32 | 3 |
| IndMDUST247_2011 | 10562_2#32 | ERR357787 | 0 | 2011 | India | 1 | 1 | 0 | 0 | 0 | 1 | 0 | 0 | 0 | 1 | 32 | 3 |
| IndMDUST248_2011 | 10492_1#43 | ERR352296 | 0 | 2011 | India | 1 | 1 | 0 | 0 | 0 | 1 | 0 | 0 | 0 | 1 | 24 | 3 |
| IndMDUST254_2010 | 10562_2#34 | ERR357789 | 0 | 2010 | India | 1 | 1 | 0 | 0 | 0 | 1 | 0 | 0 | 0 | 1 | 32 | 3 |
| IndMDUST400_2012 | 10562_2#79 | ERR357834 | 0 | 2012 | India | 1 | 1 | 0 | 0 | 0 | 1 | 0 | 0 | 0 | 1 | 24 | 3 |
| IndMDUST408_2012 | 10492_1#82 | ERR352335 | 0 | 2012 | India | 1 | 1 | 0 | 0 | 0 | 1 | 0 | 0 | 0 | 1 | 32 | 3 |
| IndSP80_2011 | 8616_4#58 | ERR420434 | 0 | 2011 | India | 1 | 1 | 0 | 0 | 0 | 1 | 0 | 0 | 0 | 1 | 32 | 3 |
| NEN_2512 | 13254_5#40 | ERR586915 | 0 | 2013 | Nepal | 1 | 1 | 0 | 0 | 0 | 1 | 0 | 0 | 0 | 1 | 32 | 3 |
| NEN_2533 | 2533_AL513382 | ERR1079310 | 0 | 2013 | Nepal | 1 | 0 | 0 | 0 | 0 | 0 | 0 | 0 | 0 | 1 | 0·38 | 2 |
| NEN_2546 | 2546_AL513382 | ERR1079311 | 0 | 2013 | Nepal | 1 | 0 | 1 | 0 | 0 | 1 | 0 | 1 | 0 | 0 | 6 | 3 |
| OTHERS_510 | 13254_5#42 | ERR586917 | 0 | 2013 | Nepal | 1 | 1 | 0 | 0 | 0 | 1 | 0 | 0 | 0 | 1 | 32 | 3 |
| OTHERS_514 | 13254_5#43 | ERR586918 | 0 | 2013 | Nepal | 1 | 0 | 1 | 0 | 0 | 1 | 0 | 1 | 0 | 0 | 8 | 3 |
| OTHERS_515 | 13254_5#44 | ERR586919 | 0 | 2013 | Nepal | 1 | 1 | 0 | 0 | 0 | 1 | 0 | 0 | 0 | 1 | 32 | 3 |
| OTHERS_516 | 13254_5#45 | ERR586920 | 0 | 2013 | Nepal | 1 | 0 | 1 | 0 | 0 | 1 | 0 | 1 | 0 | 0 | 8 | 3 |
| OTHERS_521 | 13254_5#46 | ERR586921 | 0 | 2013 | Nepal | 1 | 1 | 0 | 0 | 0 | 1 | 0 | 0 | 0 | 1 | 8 | 3 |
| OTHERS_535 | 535_AL513382 | ERR1079312 | 0 | 2013 | Nepal | 1 | 0 | 1 | 0 | 0 | 1 | 0 | 1 | 0 | 0 | 32 | 3 |
| UnkMDUST146_2012 | 10562_2#10 | ERR357765 | 0 | 2012 | Unknown | 1 | 1 | 0 | 0 | 0 | 1 | 0 | 0 | 0 | 1 | 32 | 3 |

* ciprofloxacin MIC group: 1; susceptible, 2; intermediate, 3; resistant
