# Supplementary material for: A novel ciprofloxacin-resistant subclade of H58 Salmonella Typhi is associated with fluoroquinolone treatment failure
Source: eLife. 2016 Mar 14;5:e14003. doi: 10.7554/eLife.14003 (PMC4805543; doi:10.7554/eLife.14003)
Supplement: Supplementary file 2. — (B) Table of baseline characteristics grouped by Salmonella Typhi ciprofloxacin susceptibility. (C) Table of treatment failure in detail by Salmonella Typhi lineage in the gatifloxacin treatment group. (D) Table of treatment failure in detail by ciprofloxacin susceptibility in the gatifloxacin treatment group. DOI: http://dx.doi.org/10.7554/eLife.14003.011 [file elife-14003-supp2.docx]

### Supplementary file 2A Table of Baseline characteristics by *Salmonella* Typhi lineage

| **Baseline characteristic** | **n** | **Non-H58 (N=13)** | **n** | **H58 (N=65)** | ***p* value*** |
| --- | --- | --- | --- | --- | --- |
| Age (years) – median (IQR) | 13 | 18·0(13·0,21·0) | 65 | 18·0(13·0,22·0) | 0·75 |
| Sex (male) | 13 | 12 (92·3%) | 65 | 46 (70·8%) | 0·17 |
| Temperature (°C) - median (IQR) | 13 | 39·0(38·3,39·4) | 62 | 39·0(38·3,39·4) | 0·77 |
| Days of illness before enrolment - median (IQR) | 13 | 5·0(4·0,7·0) | 65 | 5·0(4·0,7·0) | 0·39 |
| Antimicrobials in last two weeks | 13 | 1 (7·7%) | 65 | 9 (13·8%) | 1·00 |
| Previous history of typhoid | 13 | 1 (7·7%) | 65 | 5 (7·7%) | 1·00 |
| Family history of typhoid | 13 | 1 (7·7%) | 65 | 8 (12·3%) | 1·00 |
| Typhoid vaccination | 13 | 1 (7·7%) | 65 | 0 (0%) | 0·17 |
| Fever | 13 | 13 (100%) | 64 | 64 (100%) | NA |
| Cough | 12 | 4 (33·3%) | 62 | 15 (24·2%) | 0·49 |
| Constipation | 12 | 1 (8·3%) | 63 | 4 (6·3%) | 1·00 |
| Headache | 13 | 12 (92·3%) | 64 | 59 (92·2%) | 1·00 |
| Diarrhoea | 12 | 5 (41·7%) | 62 | 24 (38·7%) | 1·00 |
| Vomiting | 12 | 4 (33·3%) | 63 | 16 (25·4%) | 0·72 |
| Abdominal pain | 12 | 2 (16·7%) | 62 | 18 (29·0%) | 0·49 |
| Anorexia | 12 | 9 (75·0%) | 64 | 53 (82·8%) | 0·68 |
| Nausea | 12 | 9 (75·0%) | 61 | 38 (62·3%) | 0·52 |
| Leucocyte count (×10^9^/L) - median (IQR) | 13 | 5·9(4·5,6·6) | 65 | 6·2(4·8,7·3) | 0·54 |
| Neutrophils (%) - median (IQR) | 13 | 65·0(60,73·0) | 65 | 70(65·0,75·0) | 0·17 |
| Lymphocytes (%) - median (IQR) | 13 | 33·0(22·0,37·0) | 65 | 28·0(22·0,34·0) | 0·32 |
| Haematocrit (%) - median (IQR) | 12 | 38·7(37·1,41·2) | 65 | 38·0(35·0,41·4) | 0·39 |
| Platelet count (×10^9^/L) - median (IQR) | 13 | 177·0(165·0,189·0) | 65 | 158·0(140,210) | 0·24 |
| AST (U/L) - median (IQR) | 12 | 52·5(38·2,64·8) | 62 | 59·0(44·0,83·8) | 0·21 |
| ALT (U/L) - median (IQR) | 13 | 46·0(37·0,57·0) | 63 | 50(35·5,66·5) | 0·18 |

N refer to the number of patients in each group

n refers to the number of observations with non-missing data for the respective characteristic

*Comparisons between the two were done using Fisher’s exact test for categorical variables and the Wilcoxon rank sum test for continuous variables

### Supplementary file 2B Table of baseline characteristics grouped by *Salmonella* Typhi ciprofloxacin susceptibility

| **Baseline characteristic** | **n** | **Susceptible (N=10)** | **n** | **Intermediate (N=52)** | **n** | **Resistant (N=16)** | ***p* value*** |
| --- | --- | --- | --- | --- | --- | --- | --- |
| Age (years) – median (IQR) | 10 | 14·0(9·2,17·0) | 52 | 18·0(13·0,22·0) | 16 | 20(17·8,21·2) | 0·05 |
| Sex (male) | 10 | 8 (80%) | 52 | 39 (75·0%) | 16 | 11 (68·8%) | 0·86 |
| Temperature (°C) - median (IQR) | 10 | 39·0(38·6,39·4) | 49 | 39·0(38·3,39·4) | 16 | 38·8(38·3,39·3) | 0·42 |
| Days of illness before enrolment - median (IQR) | 10 | 4·0(4·0,6·0) | 52 | 5·0(4·0,7·0) | 16 | 5·0(4·0,7·0) | 0·88 |
| Antimicrobials in last two weeks | 10 | 1 (10%) | 52 | 7 (13·5%) | 16 | 2 (12·5%) | 1·00 |
| Previous history of typhoid | 10 | 0 (0%) | 52 | 6 (11·5%) | 16 | 0 (0%) | 0·34 |
| Family history of typhoid | 10 | 1 (10%) | 52 | 6 (11·5%) | 16 | 2 (12·5%) | 1·00 |
| Typhoid vaccination | 10 | 1 (10%) | 52 | 0 (0%) | 16 | 0 (0%) | 0·13 |
| Fever | 10 | 10 (100%) | 51 | 51 (100%) | 16 | 16 (100%) | 1·00 |
| Cough | 10 | 2 (20%) | 49 | 13 (26·5%) | 15 | 4 (26·7%) | 1·00 |
| Constipation | 10 | 0 (0%) | 50 | 4 (8·0%) | 15 | 1 (6·7%) | 1·00 |
| Headache | 10 | 10 (100%) | 51 | 45 (88·2%) | 16 | 16 (100%) | 0·34 |
| Diarrhoea | 10 | 3 (30%) | 50 | 21 (42·0%) | 14 | 5 (35·7%) | 0·82 |
| Vomiting | 10 | 2 (20%) | 50 | 14 (28·0%) | 15 | 4 (26·7%) | 0·93 |
| Abdominal pain | 10 | 1 (10%) | 50 | 16 (32·0%) | 14 | 3 (21·4%) | 0·38 |
| Anorexia | 10 | 8 (80%) | 51 | 41 (80·4%) | 15 | 13 (86·7%) | 0·91 |
| Nausea | 10 | 6 (60%) | 48 | 31 (64·6%) | 15 | 10 (66·7%) | 1·00 |

N refers to the number of patients in each group

n refers to the number of observations with non-missing data for the respective characteristic

*Comparison between the three groups were done using Fisher’s exact test for categorical variables and the Kruskal-Wallis test for continuous variables

### Supplementary file 2C Table of treatment failure in detail by *Salmonella* Typhi lineage in the gatifloxacin treatment group

| **Characteristic** | **Non-H58 (N=6)** | **H58 (N=34)** | ***p* value*** |
| --- | --- | --- | --- |
| Treatment failure | 0 (0%) | 15 (44·1%) | 0·06 |
| - Fever past seven days | 0 (0%) | 7 (21·2%) |  |
| - Rescue treatment required | 0 (0%) | 9 (27·3%) |  |
| - Microbiological failure | 0 (0%) | 2 (6·5%) |  |
| - Relapse within 28 days | 0 (0%) | 4 (14·8%) |  |
| - Confirmed relapse within 28 days | 0 (0%) | 4 (14·8%) |  |
| Any relapse during 6 month follow-up | 1 (16·7%) | 4 (11·8%) | 0·62 |
| Confirmed relapse during 6 month follow-up | 1 (16·7%) | 4 (11·8%) |  |

*Time to treatment failure and time to relapse were analyzed using Firth’s penalized maximum likelihood bias reduction method for Cox regression and *p* values were calculated from likelihood ratio tests

**Supplementary file 2D** Table of treatment failure in detail by ciprofloxacin susceptibility in gatifloxacin treatment group

| **Characteristic** | **Susceptible (N=5)** | **Intermediate (N=25)** | **Resistant (N=10)** | ***p* value*** |
| --- | --- | --- | --- | --- |
| Treatment failure | 0 (0%) | 7 (28·0%) | 8 (80%) | 0·007 |
| - Fever past seven days | 0 (0%) | 2 (8·0%) | 5 (55·6%) |  |
| - Rescue treatment required | 0 (0%) | 3 (12·0%) | 6 (66·7%) |  |
| - Microbiological failure | 0 (0%) | 0 (0%) | 2 (22·2%) |  |
| - Relapse within 28 days | 0 (0%) | 2 (9·5%) | 2 (28·6%) |  |
| - Confirmed relapse within 28 days | 0 (0%) | 2 (9·5%) | 2 (28·6%) |  |
| Any relapse during 6 month follow-up | 1 (20%) | 2 (8·0%) | 2 (20%) | 0·31 |
| Confirmed relapse during 6 month follow-up | 1 (20%) | 2 (8·0%) | 2 (20%) |  |

*Time to treatment failure and time to relapse were analyzed using Firth’s penalized maximum likelihood bias reduction method for Cox regression and *p* values were calculated from likelihood ratio tests
